# Phylogenetic Applications of the Minimum Contradiction Approach on Continuous Characters

**DOI:** 10.4137/ebo.s2505

**Published:** 2009-06-11

**Authors:** Marc Thuillard, Didier Fraix-Burnet

**Affiliations:** 1 La Colline, 2072 St-Blaise (Switzerland); 2 Université Joseph Fourier, CNRS, Laboratoire d’Astrophysique de Grenoble, BP53, F-38041 Grenoble (France). Email:thuillweb@hotmail.com

**Keywords:** phylogeny, continuous characters, minimum contradiction, galaxies, hominids

## Abstract

We describe the conditions under which a set of continuous variables or characters can be described as an X-tree or a split network. A distance matrix corresponds exactly to a split network or a valued X-tree if, after ordering of the taxa, the variables values can be embedded into a function with at most a local maximum and a local minimum, and crossing any horizontal line at most twice. In real applications, the order of the taxa best satisfying the above conditions can be obtained using the Minimum Contradiction method. This approach is applied to 2 sets of continuous characters. The first set corresponds to craniofacial landmarks in Hominids. The contradiction matrix is used to identify possible tree structures and some alternatives when they exist. We explain how to discover the main structuring characters in a tree. The second set consists of a sample of 100 galaxies. In that second example one shows how to discretize the continuous variables describing physical properties of the galaxies without disrupting the underlying tree structure.

## 1. Introduction

Maximum parsimony and distance-based approaches are the most popular methods to produce phylogenetic trees. Whereas most studies use discrete characters, there is a growing need for applying phylogenetic methods to continuous characters. Examples of continuous data include gene expressions,^1^ gene frequencies,^2^,^3^ phenotypic characters^4^ or some morphologic characters.^5^,^6^

The simplest method to deal with continuous characters using maximal parsimony consists of discretizing the characters into a number of states small enough to be processed by the software. Recent software programs such as TNT (Tree analysis using New Technology)^7^ or CoMET (Continuous-character Model Evaluation and Testing Model)^8^ use developments of the contrast method to deal with continuous characters. These methods assume that the characters evolve at comparable rates according to a Brownian motion, an assumption that is often difficult to verify.^4^,^9^ Distance-based methods are applied to both discrete and continuous input data. Compared to character-based approaches, distance-based approaches are quite fast and furnish in many instances quite reasonable results. As pointed out by Felsenstein,^9^ the amount of information that is lost when using a distance-based algorithm compared to a character-based approach is often surprisingly small. The use of continuous characters in distance-based methods may at first glance be less problematic than in character-based methods, since algorithms like the Neighbour-Joining work identically on discrete or continuous characters. However, here too it is often not easy to determine if the data can be described by a tree. When does a set of continuous characters describe a split network or an X-tree? The article furnishes some new insights on that question. It explains when a set of continuous characters can be described exactly by a split network or a valued X-tree. In real applications, the distance matrix corresponds only approximately to a split network or a tree topology. An adequate method is necessary to quantify to what extent the distance matrix corresponds to a split network or a tree. The Minimum Contradiction method can be used for that purpose.^10^–^12^

The paper is organized as follows. Section 2 succinctly presents the Minimum Contradiction method. It explains why some inequalities, called Kalmanson inequalities, are central to phylogenies. Section 3 extends the Minimum Contradiction method to a set of continuous characters. Section 4 furnishes the conditions under which a set of continuous characters can be described by a tree or a phylogenetic network. Section 5 presents an application of the algorithms in morphometrics using a set of faciocranial characters of hominids. Section 6 presents preliminary results on the evolution of a number of physical characters in galaxies. It illustrates how the Minimum Contradiction approach can be applied to discover structuring characters.

## 2. Ordering the Taxa on a Tree or a Split Network

**Figure 1:**
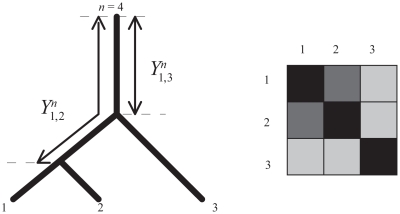
The distance *Y_i,j_^n^* ^= 4^ between a reference taxa n and the path *i*−*j* on an X-tree fulfils Kalmanson inequalities. If the values of the distance matrix *Y_i,j_^n^* ^= 4^ are coded in a gray scale, the level of gray decreases as one moves away from the diagonal. For more details see Thuillard.^10^

A valued X-tree T is a graph with X the set of leaves and a unique path between any two distinct vertices x and y, with internal vertices of at most degree 3. A circular order on an X-tree corresponds to an indexing of the n leaves according to a circular (clockwise or anti-clockwise) scanning of the leaves in T.^13^ Figure 1 shows a tree and an indexing of the taxa that corresponds to a circular order. For taxa indexed according to a circular order the distance matrix *Y_i,j_^n^* fulfils the so-called Kalmanson inequalities:^14^

(1) $$\begin{array}{l} Y_{i,j}^{n}\geq Y_{i,k}^{n},Y_{k,j}^{n}\geq Y_{k,i}^{n}(i\leq j\leq k)\;\;\;\text{with}\\ Y_{i,j}^{n}=1/2\cdot (d_{i,n}+d_{j,n}-d_{i,j}). \end{array}$$

with *d_i,j_* the pairwise distance between taxa *i* and *j*. As depicted in Figure 1, the matrix element *Y_i,j_^n^* is the distance between a reference node n and the path *i*−*j*. The diagonal elements *Y_i,i_^n^* = *d_i,n_* correspond to the pairwise distance between the reference node and the taxon *i*. The distance matrix *Y_i,j_^n^* has the property that the distance diminishes away from the diagonal.^14^ This property is visualized in Figure 1. If the values of the distance matrix are represented by different levels of gray, the level of gray is shading away from the diagonal. This property of the matrix characterizes a Kalmanson matrix and an order satisfying all Kalmanson inequalities is called a perfect order.

In real applications, the distance matrix *Y_i,j_^n^* often only partially fulfils the inequalities corresponding to a perfect order. The contradiction on the order of the taxa can be defined as

(2) $$\begin{array}{l} C=\sum_{\begin{array}{l} k>j\geq i\\ i,j,k\neq n \end{array}}{\left(\text{max}((Y_{i,k}^{n}-Y_{i,j}^{n}),0)\right)}^{2}\\ +\sum_{\begin{array}{l} k\geq j>i\\ i,j,k\neq n \end{array}}{\left(\text{max}((Y_{i,k}^{n}-Y_{j,k}^{n}),0)\right)}^{2}. \end{array}$$

The best order of a distance matrix is, by definition, the order minimizing the contradiction. The ordered matrix *Y_i,j_^n^* corresponding to the best order is defined as the minimum contradiction matrix for the reference taxon *n*. For a perfectly ordered X-tree, the contradiction *C* is zero. A high contradiction value *C* is the indication of a distance matrix deviating significantly from an X-tree. Bandelt and Dress^15^ have shown that if a distance matrix *d_i_*_,_*_j_* fulfils Kalmanson inequalities, then the distance matrix can be exactly represented by a split network or by an X-tree. A split network can be regarded as a generalization of trees. A split is a partition of the taxa into two disjoint sets that is realized by removing the edges relating the two sets. (For an introduction to split networks, see).^16^ Kalmanson inequalities are related to a number of interesting mathematical results. Kalmanson inequalities relate phylogenetic trees and split networks to the travelling salesman problem. Let us recall that the travelling salesman problem is a fundamental problem in computer science. The problem’s formulation is quite simple. A travelling salesman must visit a number of cities and return to its point of departure. The problem consists of finding the order of the cities that minimizes the total travelling distance $D=d_{n,1}+\sum_{i=1,\ldots ,(n-1)}d_{i,i+1}$ with *d_i_*_,_*_j_* the distance between the city *i* and *j*. The travelling salesman is one of the most studied problem in computational science as it is the prototype of a difficult problem. For all known algorithms, the maximum computing time to solve the travelling salesman problem increases very rapidly with the number of cities. In other words, the solution of the travelling salesman problem for a large number of cities generally requires a very large computing power. Already for a few hundreds cities, only approximate solutions can be obtained by the largest computers. Not all TSP problems are difficult to solve. For instance, the TSP is easy to solve when the cities are on a convex hull in the Euclidean plane. In order to be on a convex hull, the cities must be orderable so that the following inequalities hold: *d_i,j_* +*d_k,n_* ≤ *d_i,k_* + *d_j,n_* and *d_i,n_* + *d_j,k_* ≤ *d_i,j_* + *d_k,n_* with 1≤ *i* ≤ *j* ≤ *k* ≤ *n*.^14^ These inequalities are equivalent to the Kalmanson inequalities (1): *Y_i,j_^n^* ≥ *Y_i,k_^n^*; *Y_k,j_^n^* ≥ *Y_k,i_^n^* (*i* ≤ *j* ≤ *k* ≤ *n*). The solution to the TSP corresponds to the order of the cities on the convex hull.

If one leaves aside Euclidian geometry, other metrics fulfil Kalmanson inequalities. Kalmanson inequalities are also satisfied by taxa on an X-tree or a split network. If the taxa are circularly ordered, then the Kalmanson inequalities are fulfilled. As developed in a number of publications,^17^–^19^ perfect order corresponds in X-trees and split networks to a solution of the travelling salesman problem (TSP) for both the distance matrices *d_i_*_,_*_j_* and *Y_i,j_^n^*.

In the next section we show that for trees and split networks as well, the Kalmanson inequalities are related to convexity. This result furnishes a new perspective on when trees and phylogenetic networks can be used to describe a set of continuous characters.

## 3. Kalmanson Inequalities on a Single Continuous Character

As of today, it is still not really clear when the use of continuous characters in distance-based phylogenetic studies is a valid approach. To clarify that problem, we will first consider a single character.

Let us now discuss the conditions for which a set of taxa characterized by a single continuous character *f*_1_ can be perfectly ordered. Let us define the distance *d_i,j_* between two taxa as *d_i,j_* = *abs*(*f* (*i*) − *f* (*j*)). The taxa {1, …, *n*} are perfectly ordered when the order is such that the distance matrix *Y_i,j_^n^* fulfils the Kalmanson inequalities: *Y_i,j_^n^* ≥ *Y_i,k_^n^* *Y_k,j_^n^* ≥ *Y_k,i_^n^* (1≤ *i* ≤ *j* ≤ *k* ≤ *n*). Proposition 1 describes the necessary and sufficient conditions on the character *f*_1_(*i*) so that the taxa can be perfectly ordered.

### Proposition 1

A distance matrix *Y_i,j_^n^* is Kalmanson if and only if the values *f*_1_(*i*) of a character on an ordered set of taxa can be embedded into a continuous function *f*(*x*) on [1,*n*]: *f*(*x*) = (*x*−*i*) · (*f*(*i*+1) − *f*(*i*)) + *f*(*i*), *x* ∈ [*i*,*i* + 1], *x* ⊂ ℜ, *i* ∈ {1, …, *n*} with the following properties:

- the function *f* (*x*) has at most one local maximum and one local minimum
- the function *f* (*x*) crosses the reference line *L*(*x*) = *f*_1_(*n*) = *const* at most once.

### Proof

A central distinction can be made between the taxa depending on whether the character value is smaller or larger than the value of a reference taxon *n*. The set of taxa can be divided into two disjoint sets, the set *S* of taxa with values smaller or equal to the reference value *f*_1_(*n*) and the set of taxa *L* with values larger than the reference value (See Fig. 5 for an illustration). Let us show that a distance matrix fulfilling the conditions i) and ii) is perfectly ordered for any 3 ordered taxa *i* ≤ *j* ≤ *k*. We will consider all possible cases.

- All 3 taxa are in the same set (*S* or *L*). The distance *Y_i,j_^n^* between the taxa *i* and *j* is given by the expression *Y_i,j_^n^* = min(|*f*_1_(*i*) − *f*_1_(*n*)|,|*f*_1_(*j*) − *f*_1_(*n*)|). Under the conditions in Prop. 1 one has min(|*f*_1_(*i*) − *f*_1_(*n*)|, |*f*_1_(*j*) − *f*_1_(*n*)|) ≥ min(|*f*_1_(*i*) − *f*_1_(*n*)|,|*f*_1_(*k*) − *f*_1_(*n*)|) and consequently *Y_i,j_^n^*≥ *Y_i,k_^n^*, (*i* ≤ *j* ≤ *k* ≤ *n*).
- The taxon i is in one set of taxa and the taxa *j*, *k* in another set. In that case one has *Y_i,j_^n^* = *Y_i,k_^n^* = 0. (For an illustration, see Fig. 5 and Eq. 3)
- Condition ii) prevents the second taxon to be in another set than the taxa *i* and *k*.
- If the third taxa is in another set than the taxa *i*, *j* one has *Y_i,j_^n^* ≥ *Y_i,k_^n^* = 0. The proof for the second inequality *Y_k,j_^n^* ≥ *Y_k,i_^n^* (*i* ≤ *j* ≤ *k* ≤ *n*) is similar.

Let us show that if the conditions of the proposition are not fulfilled then Kalmanson inequalities are violated. If the function *f*(*x*) has two maxima (or 2 minima) corresponding to the taxa *i* and *k*, then there exists a taxa *j* with *Y_i,j_^n^* < *Y_i,k_^n^* and consequently the Kalmanson inequalities are not fulfilled. A similar inequality holds if the function *f*(*x*) does not satisfy condition ii).

Figure 3 illustrates Prop. 1 with a simple example. The matrix *Y_i,j_^n^* is depicted using a colour coding. Large values are coded red, while small values of *Y_i,j_^n^* correspond to small values. The distance matrix is perfectly ordered; the values of *Y_i,j_^n^* decrease away from the diagonal as prescribed by the Kalmanson inequalities. Two clusters are observed, the first cluster corresponds to values smaller than the reference value, the second cluster to values larger than the reference value.

The results on a single character can be easily generalized to several characters as the sum of perfectly ordered matrices $Y_{i,j}^{n}=\sum_{m=1}^{m\text{max}}Y_{i,j}^{n}(f_{m})$ is also perfectly ordered. This follows directly from the Kalmanson inequalities. If each character is Kalmanson, then *Y_i,j_^n^*(*f_m_*)≥ *Y_i,k_^n^*(*f_m_*) and *Y_k,j_^n^*(*f_m_*) ≥ *Y_k,i_^n^*(*f_m_*) (*i* ≤ *j* ≤ *k* ≤ *n*), and therefore *Y_i,j_^n^*is perfectly ordered.

**Figure 2:**
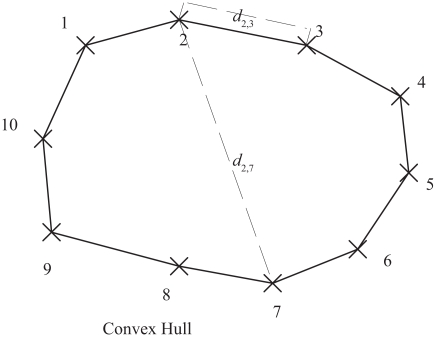
The travelling salesman problem (TSP) can be easily solved if the points are on a convex hull in the Euclidean plane. Points on a convex hull fulfil the Kalmanson inequalities.

We are now ready to discuss the connection between Kalmanson inequalities and convexity in phylogenies. The tree metrics case is different from the Euclidean metrics described in Figure 2. In an Euclidean metrics, Kalmanson inequalities are fulfilled if the points (cities) are on a convex hull, while for split networks and trees the hull must be orthogonally convex. In an Euclidean metrics, a set *Z* ⊂ ℜ*^n^* is defined to be orthogonally convex if, for every line that is parallel to one of the axes of the Cartesian coordinate system, the intersection of *Z* with the line is empty, a point, or a single interval.

### Corollary 2

If the taxa {1, …, *n*} are ordered so that the distance matrices *Y_i,j_^n^* associated to the 2 characters *f*_1_ and *f*_2_ are perfectly ordered, then the closed circuit {(*f*_1_(1), *f*_2_(1), …, (*f*_1_(*n*), *f*_2_(*n*)} relating each two consecutive points by an edge is on an orthogonal convex hull.

### Proof

**Figure 3:**
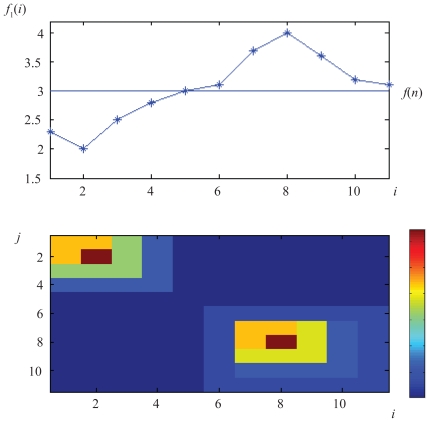
Top: The taxa are ordered so that the characters *f*_1_(*i*) on the taxa {1, …, *i*, …, *n*} can be embedded in a function *f*(*x*) fulfilling proposition 1. Bottom: Distance matrix *Y_i,j_^n^* with a colour coding. Larger values are coded red, small values blue. The order is perfect (*C* = 0 in Eq. 2).

Proposition 1 for a single character is equivalent to the following proposition: if the distance matrix *Y_i,j_^n^* associated to a character *f*_1_ is Kalmanson, then any horizontal line crosses the function *f*(*x*) at most once (see Fig. 3 for an illustration). It follows that any horizontal or vertical line in the Euclidian plane intersects the closed curve {(*f*_1_(1), *f*_2_(1), …, (*f*_1_(*n*), *f*_2_(*n*)} at most twice. (The intersection of the line with *Z* is either a single interval or a point or empty (no crossing)). Let us point out that Corollary 2 describes a sufficient but not necessary condition to obtain a perfectly ordered matrix *Y_i,j_^n^*.

Corollary 2 can be extended to higher dimensions. The geometry, associated to trees and split networks built on a set of perfectly ordered characters, corresponds to an orthogonally convex hull.

## 4. How to Build a Tree or a Phylogenetic Network from Single Continuous Characters?

In the previous section we have explained when a set of characters on a set of taxa fulfils Kalmanson inequalities and can be described by a tree or a split network. In this section, we explicitly show how the branches of the trees evolve when several characters are combined. For a single character, the taxa can be ordered so as to fulfil the conditions of Prop. 1. The resulting tree is a line tree. In a line tree, all taxa are on a single path and one has

(3) $$\begin{array}{l} 0\;\;\;i\in S,j\notin S\;\;\;\text{or}\;\;\;i\in L,j\notin L\\ Y_{i,j}^{n}=\text{min}(|f(i)-f(n)|,|f(j)-f(n)|)\\ =\text{min}(Y_{i,i}^{n},Y_{j,j}^{n})\;\;\;otherwise \end{array}$$

**Figure 5:**
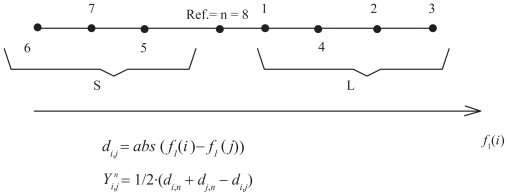
The tree associated to a single character is a line tree. In a line tree, all taxa are on the same path.

Figure 5 shows an example of a line tree with perfectly ordered taxa.

At least two independent characters are necessary to generate a tree that is not a line tree. An independent character can be defined as follows.

### Definition 1

Two characters *f*_1_ and *f*_2_ are independent if there exists at least 2 taxa *i* and *j* (*i* < *j* < *n*) so that 0 < *Y_i,j_^n^* < *Y_i,i_^n^*, *Y_j,j_^n^* with *Y_i,j_^n^* = *Y_i,j_^n^*(*f*_1_) + *Y_i,j_^n^*(*f*_2_).

**Figure 6:**
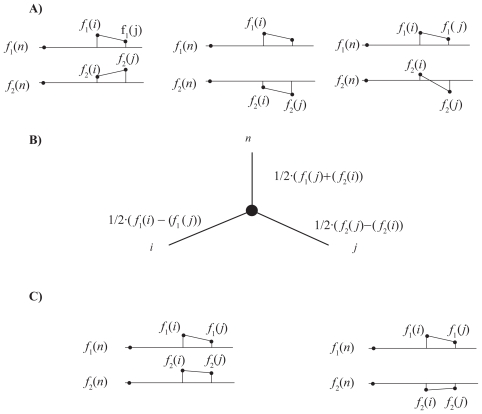
**A**) Examples of independent characters, **B**) X-tree corresponding to the first two examples, **C**) The characters *f*_1_ and *f*_2_ are not independent.

Figure 6a shows 3 examples of independent characters. If two characters are independent and the taxa are perfectly ordered on both *f*_1_ and *f*_2_, then the distance matrix corresponds to a split network or an X-tree different from a line tree. Let us discuss the first example in Figure 6. Without restriction, let us assume that for the reference taxon *n*, *f*_1_(*n*) = *f*_2_(*n*) = 0. The distance matrix elements are given by

$$Y_{i,j}^{n}=\left(\begin{array}{cc} f_{1}(i)+f_{2}(i) & \text{min}(f_{1}(i),f_{1}(j))+\text{min}(f_{2}(i),f_{2}(j))\\ \text{min}(f_{1}(i),f_{1}(j))+\text{min}(f_{2}(i),f_{2}(j)) & f_{1}(j)+f_{2}(j) \end{array}\right).$$

#### Proposition 3

If two characters *f*_1_ and *f*_2_ are independent, then the distance matrix *Y_i,j_^n^* = *Y_i,j_^n^*(*f*_1_) + *Y_i,j_^n^*(*f*_2_) does not correspond to a line tree.

### Proof

A line tree is so that either *Y_i,j_^n^* = 0 or *Y_i,j_^n^* = min (*Y_i,i_^n^*, *Y_j,j_^n^*). By definition two independent characters do not fulfil either equality.

The expression reduces to $Y_{i,j}^{n}=\left(\begin{array}{ll} f_{1}(i)+f_{2}(i) & f_{1}(j)+f_{2}(i)\\ f_{1}(j)+f_{2}(i) & f_{1}(j)+f_{2}(j) \end{array}\right)$ and one has 0 < *Y_i,j_^n^* < *Y_i,i_^n^*, *Y_j,j_^n^*. The distance matrix describes the X-tree in Figure 6b. Two examples of characters that are not independent are given in Figure 6c.

**Figure 7:**
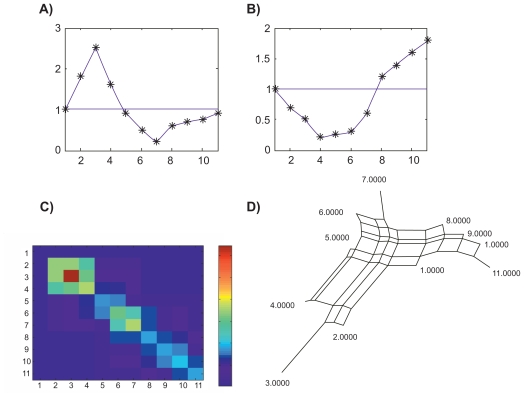
The distance matrix *Y_i,j_^n^* (Fig. 7c) corresponding to two dependent characters *f*_1_(*i*) and *f*_2_(*i*) (Fig. 7a,b). The distance matrix corresponds to a split network (Fig. 7d). The split network is obtained with Splits Tree.^16^ The contradiction on the order of the taxa is zero (*C* = 0 in Eq. 2)

Figure 7 is another illustration of Proposition 3 for two characters on perfectly ordered taxa. The ordered matrix *Y_i,j_^n^* = *Y_i,j_^n^*(*f*_1_) + *Y_i,j_^n^*(*f*_2_) is perfectly ordered. In this example, the distance matrix is described by a split network and not by an X-tree (A tree is a special case among split networks).^10^

## 5. Classification of Hominids Fossil Specimens

The Minimum Contradiction on continuous characters was tested on a set of independently analyzed data representing craniofacial properties of hominid fossils. The results obtained with the Minimum Contradiction Method are compared to those obtained with TNT in a recent article in Nature. González-José et al^6^ have analysed sets of craniofacial landmarks representing the flexure of the cranial base, facial retraction, neurocranial globularity, and masticatory apparatus. Phylogenetic relationships among *Homo* species and hominid taxa were obtained with the maximum parsimony module for continuous characters in TNT. The reader is referred to González-José et al^6^ for the details on the extraction of the data.

**Figure 8:**
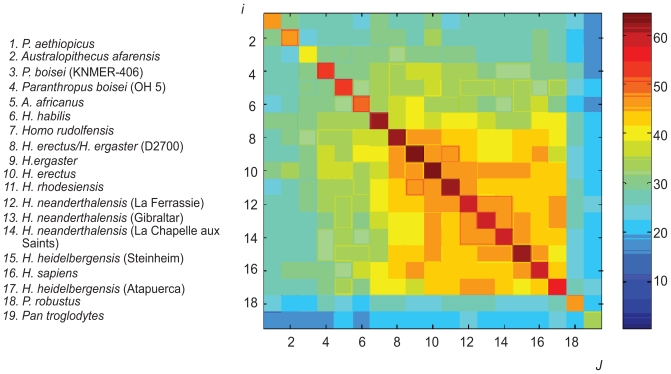
Minimum contradiction matrix *Y_i,j_^n^* on a set of 20 hominid taxa using *Gorilla gorilla* as reference taxon *n*.

Similarly to González-José et al, we have preprocessed the 4 sets of landmarks with the Generalized Procrustes Analysis in Morphologika.^20^ The Generalized Procrustes analysis is a superimposition method that rotates, scales and translates the landmarks to adjust for isometric effects of size and orientation. The distance between two taxa is computed as the sum of the absolute difference between each Procrustes coordinate. The best circular order was subsequently obtained by minimizing the contradiction *C* in Eq. (1).^11^ Figure 8 shows the minimum contradiction matrix using *Gorilla gorilla* as reference taxon. *Gorilla gorilla* is taken as the reference taxon in order to be able to compare the results with González-José et al.

The matrix *Y_i,j_^n^* is depicted using a colour coding. Large values are coded red, while blue corresponds to small values of *Y_i,j_^n^*. The minimum contradiction matrix can be described as a split network. The order of the taxa is quite compatible with the maximum parsimony tree of González-José et al. A number of contradictions to perfect order are observed for instance *H. sapiens* vs. *H. ergaster*. As an example, let us describe how the contradiction between *H. sapiens* and *H. ergaster* can be extracted from Figure 8. The value *Y*_9,16_*^n^* is coded in orange (45 on the right scale). The element *Y*_9,16_*^n^* is larger than for instance *Y*_9,13_*^n^* (Yellow = 41) or *Y*_14,16_*^n^* = 42. This corresponds to a contradiction as according to the Kalmanson inequalities, one should have *Y*_9,16_*^n^* ≤ *Y*_9,13_*^n^* and *Y*_9,16_*^n^* ≤ *Y*_14,16_*^n^*. Contradictions in *Y_i,j_^n^* correspond to deviations from a tree or a split network structure possibly caused by homoplasies or lateral transfers in genetic sequences.^11^

Table 1 shows the best order obtained with the minimum contradiction approach and the order of the taxa on the maximum parsimony tree. (The best order is a circular order and *Gorilla gorilla* is adjacent to both *P. aethiopicus* and *Pan troglodytes*.) Except for *H. sapiens* the specimens are very similarly ordered. The 2 main branches of the maximum parsimony tree are indicated by a colour in the Table 1.

Let us illustrate with an example the possibilities offered by the Minimum Contradiction Method to analyze phylogenetic data. In Figure 8, the largest values of *Y_i,j_^n^* for *i* = *H. habilis* and *H. rudolfensis* correspond to *j* = *H. ergaster* and *H. sapiens* (*Y_i,j_^n^*: yellow = 41). Grouping *H. habilis* and *H. rudolfensis* with the other *Homo* taxa is therefore a possibility. On the other hand *Y_i,j_^n^* has comparable values within the cluster *H. habilis, H. rudolfensis, A. africanus, P. boisei* (KNMER-406), and *Paranthropus boisei* (OH 5). This offers a second interpretation, namely that *H. habilis* and *H. rudolfensis* are related to non *Homo* taxa. In order to proceed with the analysis, some definitions have to be introduced. Two consecutive taxa with different character values define a cut. Two cuts in a circular order define a split. A character is said to support a set of splits, corresponding to all possible pairs of cuts, if after discretization of the character’s values the taxa are perfectly ordered. (As a side remark, let us mention the connection existing between the definition of a continuous character supporting a split and the convexity of character states in a (non-valued) X-tree. If a character supports a split on a valued X-tree then the character states after discretization are convex).^21^

**Figure 9:**
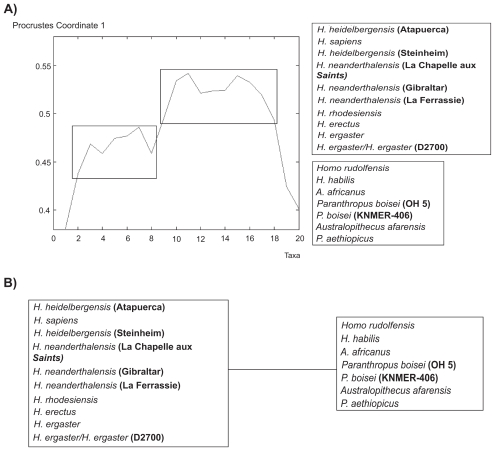
Examples showing how characters supporting well a split can be identified using Prop. 1 in this article. The order is the same as in Table I. **A**) The character “Facial retraction: landmark 9” supports the split between *Homo* without *H. habilis* and *H. rudolfensis* and the other taxa. **B**) Split for the character “Facial retraction: landmark 9”.

Contrarily to González-José et al our analysis is done without using a Principal Components Analysis (PCA). This simplifies considerably the interpretation of the results. Landmarks satisfying to a good approximation Prop. 1 can be identified quite simply. Once those characters are identified, one can discover which splits are supported by each character. Figure 9 shows a character that supports the second interpretation of Figure 8. The landmark 9 (Facial retraction) supports a split between *Homo* without *H. habilis* and *H. rudolfensis* and the other taxa. In that example, both interpretations are equally valid.^22^

**Figure 10:**
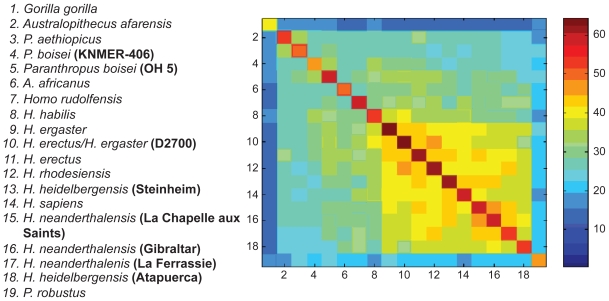
Minimum contradiction matrix *Y_i,j_^n^* on a set of 20 hominid taxa using *Pan troglodytes* as reference taxon *n*.

The level of contradiction can be used as an objective criterion to choose the reference node. As discussed in details in Thuillard,^11^,^12^ the reference node is an important choice in the presence of contradictions. In our example, the normalized level of contradiction is about 30% lower with *Pan troglodytes* as reference taxon. This suggests that *Pan troglodytes* is a better choice than *Gorilla gorilla* as a reference taxon. Figure 10 shows quite interestingly that the ambiguity concerning *H. habilis* is removed with *Pan troglodytes* as reference taxon. *H. habilis* belongs clearly to *Homo*. In summary, with the data analyzed here, *H. habilis* shares some characters with non *Homo*, but has a majority of characters shared with other *Homo* specimen, predominantly *H. erectus/H. ergaster*.

A deeper analysis of the above results would go much beyond the goal of this section. In this section we wanted to illustrate how information can be extracted from a minimum contradiction analysis on continuous variables.

## 6. Galaxies

The second example, illustrating the continuous minimum contradiction approach, shows how a character-based phylogenetic tree can be inferred from a distance matrix. A standard approach to constructing phylogenetic trees from continuous variables consists of discretizing the variables and to run a maximum parsimony software treating the discretized variables as characters. The difficulty with that approach is that the discretization may easily disrupt an underlying tree structure. This problem is particularly acute when 2-states characters are used. The Minimum Contradiction Method can be applied to remedy that problem. For illustration, we have taken from Ogando et al^23^ a sample of 100 galaxies described by some observables and derived quantities. In this section, our goal is to illustrate how the Minimum Contradiction approach can be used in practice, in particular to discover structuring characters. The astrophysical implications are out of the scope of the present work. It will be presented in subsequent papers together with more in-depth analysis. In practice, identifying a priori characters that behave like on Figure 7a is difficult. For complex objects in evolution, this would require some good knowledge of the evolution of the characters together with some ideas about the correct phylogeny or at least a rough evolutionary classification. In astrophysics, the study of galaxy evolution has not yet reached this point.^24^–^27^ However, we want to show here how the approach presented in this paper can be extremely valuable even in cases with very little a priori hints.

**Figure 11:**
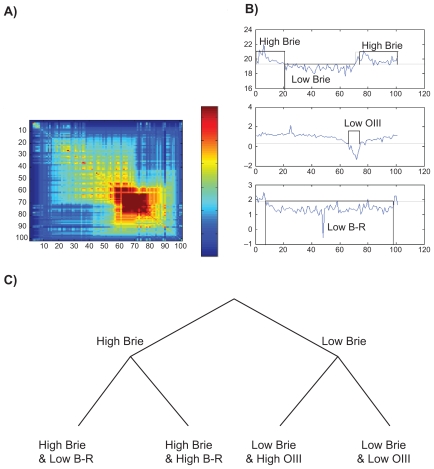
Analysis of 3 selected characters Brie, OIII and B–R on an ensemble of 100 galaxies ordered with the Minimum Contradiction method. **A**) Distance matrix *Y_i,j_^n^*; **B**) Character values vs. Galaxies after ordering: Top character Brie, Middle: character OIII, Bottom: Character B–R; **c**) Tree describing approximately the distance matrix after discretization (Solid line in b).

In this example, three variables are selected: Brie, B–R, and OIII. Brie measures the surface brightness of the galaxy, on a negative logarithm scale. B–R is the difference between the B- and R-magnitudes: a high B–R indicates a red object (old stars and/or high metallicity), while a low B-R indicates a blue object (young stars and/or low metallicity). There is no a priori direct physical connections between the three variables. High OIII (star formation) could be expected to correspond to low B–R (young stars). As shown in Figure 11, that is not always true, due in large part to the dependence of B–R on the metallicity of the stars.

After ordering, a number of clusters are clearly recognized. The galaxies associated to the discrete character “High Brie” are far from being perfectly ordered. The data cannot be described well with either a split network or a tree. This problem can be solved by discretizing the variables. In Figure 11b, the 3 ordered variables are represented together with a discretization of the input variable using threshold values (dashed lines). Discretization removes most contradictions on the order (In order to see it, let us consider the character Brie. Let us code Brie High as 1 and Brie low as 0. The discretized function fulfils Prop. 1 as it has only a minimum and any horizontal line crosses the discretized function at most twice). The distance matrix corresponds well to a split network. The split network can be represented, in first approximation, by an X-tree. To do so let us move the boundary (dashed line) separating “low” from “high Brie” slightly to the right. The main split in the tree corresponds to the “High Brie” and “Low Brie” branches. Each branch is split into two other branches defined by the character states, “low OIII”, “High OIII” for “Low Brie” and “low B–R”, “High-B–R” for “High-Brie”. The resulting tree is shown in Figure 11c.

The main splitting character is Brie for which our discretization separates our sample in two roughly equal bins. That is not the case for OIII and B-R for which low OIII and high B–R are two small and distinct groups. All high Brie galaxies are in the high OIII bin. Indeed, a low OIII corresponds to an absorption feature, while a high OIII indicates an emission line due to star formation. As a consequence, in this limited sample, low surface brightness galaxies (main left branch) do have star formation, and some high surface brightness objects show only an OIII absorption feature (rightmost branch). All high B–R galaxies have high Brie and high OIII. This means that in this sample, the red objects have a low surface brightness, but they have some star formation. They are thus not simply ageing galaxies, but probably form stars with high metallicity. Conversely, all low OIII galaxies of our sample have a low B–R, so that blue objects do not necessarily form a lot of stars.

A better understanding of the groupings and their physical implications would require the investigation of other properties of the objects. The relative complexity of the correlations between our three characters implies that a correct classification cannot be made by dichotomizing the variables beforehand. A more objective and multivariate point of view is necessary to precise the separating value between for instance “high” and “low” as in our present study. Indeed, the discretization is here used only to depict more easily the multivariate and continuous ordering of the objects in the sample. Figure 11c is a synthetic classification shown by the distance matrix 11b and obtained from the Minimum Contradiction method using fully continuous information.

## 7. Conclusions

The Minimum Contradiction approach furnishes an objective justification to using continuous variables or characters in phylogenetic studies. Provided the taxa can be ordered so that each character fulfils the Kalmanson inequalities then there exists a split network or a tree representing exactly the distance matrix. We have shown that the Kalmanson inequalities are fulfilled if the values of each character can be embedded into a function with at most a local maximum and a local minimum, and crossing any horizontal line at most twice. In practical applications the level of contradiction of the minimum contradiction matrix furnishes an objective measure of the deviations to a tree or split network. This approach was applied to a set of continuous characters, representing faciocranial landmarks of hominids, already analyzed with a maximum parsimony approach.^6^ While the results are found to be very similar to the maximum parsimony approach, the Minimum Contradiction method furnishes supplementary information: i) Problematic relationships between taxa are visualized. ii) Characters supporting quite well a split can be discovered as they correspond to single characters fulfilling very well the Kalmanson inequalities. iii) Our approach can also select the best outgroup (reference taxon). The best outgroup leads to the order with the smallest level of contradiction.

Discovering the structuring characters among a set of continuous characters is a notoriously difficult task. The search for structuring characters can be greatly facilitated by looking for subsets of characters that satisfy best the Kalmanson inequalities. This approach was applied to a set of 40 characters on 100 galaxies to extract the structuring characters. Quite interestingly, while discretization of continuous characters is often problematic, discretization with the Minimum Contradiction method can help removing contradictions from a split network or tree structure.

## Figures and Tables

**Figure 4:**
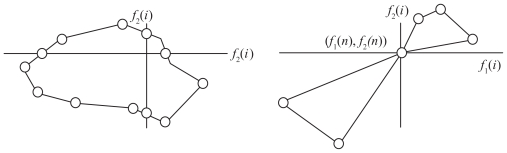
The values of two characters that are perfectly ordered are on an orthogonal convex hull. Two examples of an orthogonal convex hulls.

**Table 1:** Circular order obtained with the Minimum Contradiction and the Maximum Parsimony approach on a set of craniofacial landmarks of hominids (Maximum Parsimony order adapted from González-José et al).^6^

Minimum contradiction	Maximum parsimony
*0. Gorilla gorilla*	*Gorilla gorilla*
*1. P. aethiopicus*	*P. aethiopicus*
*2. Australopithecus afarensis*	*Australopithecus afarensis*
*3. P. boisei* (KNMER-406)	*P. boisei* (KNMER-406)
*4. Paranthropus boisei* (OH 5)	*Paranthropus boisei*
*5. A. africanus*	*A. africanus* (OH 5)
*6. H. habilis*	*H. habilis*
*7. Homo rudolfensis*	*Homo rudolfensis*
*8. H. erectus/H. ergaster* (D2700)	*H. erectus/H. ergaster* (D2700)
*9. H. ergaster*	*H. ergaster*
*10. H. erectus*	*H. erectus*
*11. H. rhodesiensis*	*H. rhodesiensis*
*12. H. neanderthalensis* (La Ferrassie)	*H. sapiens*
*13. H. neanderthalensis* (Gibraltar)	*H. neanderthalensis* (La Ferrassie)
*14. H. neanderthalensis* (La Chapelle aux Saints)	*H. neanderthalensis* (La Chapelle aux Saints)
*15. H. heidelbergensis* (Steinheim)	*H. neanderthalensis* (Gibraltar)
*16. H. sapiens*	*H. heidelbergensis* (Atapuerca)
*17. H. heidelbergensis* (Atapuerca)	*H. heidelbergensis* (Steinheim)
*18. P. robustus*	*P. robustus*
*19. Pan troglodytes*	*Pan troglodytes*
